# Observational study on efficacy of negative expiratory pressure test proposed as screening for obstructive sleep apnea syndrome among commercial interstate bus drivers - protocol study

**DOI:** 10.1186/1471-2466-11-57

**Published:** 2011-12-07

**Authors:** Raquel P Hirata, Isabella C Aguiar, Sergio R Nacif, Lilian C Giannasi, Fernando SS Leitão Filho, Israel R Santos, Salvatore Romano, Newton S Faria, Paula N Nonaka, Luciana MM Sampaio, Claudia S Oliveira, Paulo TC Carvalho, Geraldo Lorenzi-Filho, Alberto Braghiroli, Adriana Salvaggio, Giuseppe Insalaco, Luis VF Oliveira

**Affiliations:** 1 Master's and Doctoral Degree Programs in Rehabilitation Sciences, Nove de Julho, University, Sao Paulo, Brazil; 2 Pulmonary Division, Servidor Publico do Estado de Sao Paulo Hospital, Sao Paulo, Brazil; 3 School of Dentistry, Julio Mesquita Filho State University, Sao Jose dos Campos, Brazil; 4 Department of Medicine, Fortaleza University, Fortaleza, Brazil; 5 National Research Council of Italy, Institute of Biomedicine and Molecular Immunology "A. Monroy", Palermo, Italy; 6 Sleep Laboratory, Pulmonary Division, Heart Institute (InCor) Sao Paulo University, Medicine School, Sao Paulo, Brazil; 7 Sleep Laboratory, Medical Center of Veruno, Salvatore Maugeri Foundation, Veruno (NO) Italy

## Abstract

**Abstract:**

**Background**

Obstructive sleep apnea (OSA) is a respiratory disease characterized by the collapse of the extrathoracic airway and has important social implications related to accidents and cardiovascular risk. The main objective of the present study was to investigate whether the drop in expiratory flow and the volume expired in 0.2 s during the application of negative expiratory pressure (NEP) are associated with the presence and severity of OSA in a population of professional interstate bus drivers who travel medium and long distances.

**Methods/Design**

An observational, analytic study will be carried out involving adult male subjects of an interstate bus company. Those who agree to participate will undergo a detailed patient history, physical examination involving determination of blood pressure, anthropometric data, circumference measurements (hips, waist and neck), tonsils and Mallampati index. Moreover, specific questionnaires addressing sleep apnea and excessive daytime sleepiness will be administered. Data acquisition will be completely anonymous. Following the medical examination, the participants will perform a spirometry, NEP test and standard overnight polysomnography. The NEP test is performed through the administration of negative pressure at the mouth during expiration. This is a practical test performed while awake and requires little cooperation from the subject. In the absence of expiratory flow limitation, the increase in the pressure gradient between the alveoli and open upper airway caused by NEP results in an increase in expiratory flow.

**Discussion**

Despite the abundance of scientific evidence, OSA is still underdiagnosed in the general population. In addition, diagnostic procedures are expensive, and predictive criteria are still unsatisfactory. Because increased upper airway collapsibility is one of the main determinants of OSA, the response to the application of NEP could be a predictor of this disorder. With the enrollment of this study protocol, the expectation is to encounter predictive NEP values for different degrees of OSA in order to contribute toward an early diagnosis of this condition and reduce its impact and complications among commercial interstate bus drivers.

**Trial registration:**

*Registro Brasileiro de Ensaios Clinicos *(local acronym RBEC) [Internet]: Rio de Janeiro (RJ): *Instituto de Informaçao Cientifica e Tecnologica em Saude *(Brazil); 2010 - Identifier RBR-7dq5xx. Cross-sectional study on efficacy of negative expiratory pressure test proposed as screening for obstructive sleep apnea syndrome among commercial interstate bus drivers; 2011 May 31 [7 pages]. Available from http://www.ensaiosclinicos.gov.br/rg/RBR-7dq5xx/.

## Background

Sleep disorders are common throughout the world and have living effects on modern industrialized "24-hour" societies. The consequences of such disorders include excessive sleepiness, a negative effect on social and recreational activities, a decreased physical ability, a decline in productivity and a high risk of accidents [1]. These conditions, which are associated to acute or chronic insomnia, chronic sleep restriction, work shifts, jet lag, narcolepsy and obstructive sleep apnea (OSA), are a public health concern.

There is a large body of evidence demonstrating that sleepiness contributes toward industrial and traffic accidents [2]. It has previous been reported that 22% of accidents are caused by excessive sleepiness and 17-19% of traffic deaths are the result of sleepiness at the wheel [3]. The medical and economic costs of traffic accidents are estimated to be 1 to 3% of the gross domestic product of a country (annual cost of approximately 518 billion dollars). The *Instituto de Pesquisa Econômica Aplicada *(IPEA) [Institute of Applied Economic Research] of the Brazilian Federal Government reports that the mean cost of traffic accidents in Brazil is US$ 5,167,000, among which US$ 1,919.000 are spent on victimless accidents, US$ 2,942,000 are spent on accidents that result in injuries and US$ 2,476,000 are spent on accidents involving deaths [4].

OSA is a respiratory disease characterized by the collapse of the upper airways which occurs during sleep in predisposed subjects. Following chronic obstructive pulmonary disease and asthma, OSA is the epidemiological most important and widespread respiratory disease, affecting 3 to 7% of the male population and 2 to 5% of the female population between 40 and 65 years of age in the western world [5]. In Brazil, prevalence even larger was encountered according to an epidemiological study carried out in the city of Sao Paulo, where 24.8% males and 9.6% females were OSA patients [6].

One of the most important social implications of OSA is the increased risk for driving accidents [7]. Pack et al. (2002) reported a prevalence of 28% in a population of professional truck drivers [8]. The main cause of road accidents among professional drivers is lack of sleep, disturbance in the sleep/wake cycle (shift workers) and sleep disorders [9,10].

There is a close relationship between OSA and the risk of cardiovascular disease [11-13], neuropsychological problems [14,15], reduction in quality of life [16,17] and increase in the use of health resources [18,19], demonstrating that, when under-diagnosed, OSA can have serious consequences. Thus, the identification of new markers for OSA can be of considerable relevance for clinical practice.

An increase in the upper airway collapsibility is one of the main determinants of OSA [20,21]. Investigators have identified anatomical factors [22-24], neuromuscular control factors [25,26] and liquid and fat deposits [27] that may lead to increased pharyngeal collapsibility during sleep.

The negative expiratory pressure (NEP) method was initially used to assess intrathoracic expiratory flow limitations in patients with chronic obstructive pulmonary disease [28]. However, NEP test in individuals with OSA was found to lead to a collapse of the extrathoracic airway, with a drop in expiratory flow below the preceding expiration, which is common among patients with OSA [29-32]. The NEP test is carried out by administering negative pressure at the mouth during expiration. This maneuver is easy to perform and requires minimal patient cooperation. NEP test is based on the pressure gradient increase between the alveoli and open upper airway that results in an increase in expiratory flow. This study will allow the use of new screening technique to detect upper airway collapsibility, a high risk indicator for OSA in professional drivers. It's a daytime, low-cost screening method that could be included in a preventive strategy aimed at reducing the impact of this condition on accidents and the development of cardiovascular consequences.

### Objectives

The main objective of the study, which will be conducted on medium and long distance professional interstate bus drivers, is to investigate whether NEP test induced upper airway collapse is indicative for the presence and severity of OSA. Secondary objectives are: 1) identify the prevalence of OSA in this population; 2) determine the prevalence of metabolic syndrome and cardiovascular disease; 3) determine the correlation of work shift on clinical findings; and 4) validate a novel questionnaire on sleep quality proposed by the *Italian National Research Council Institute of Biomedicine and Molecular Immunology "A. Monroy" *and *Centro Medico di Veruno *(Italy).

## Methods/Design

### Study design

**Figure 1:**
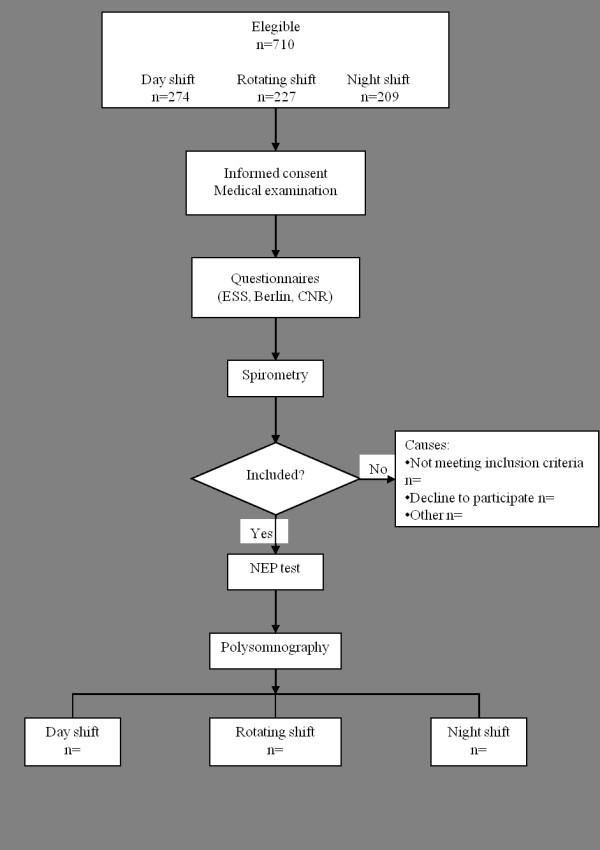
**Design of the study**. Abbreviations: ESS - Epworth Sleepiness Scale; CNR - Consiglio Nazionale delle Ricerche; NEP - negative expiratory pressure.

An observational, analytical study will be carried out at the Sleep Laboratory of the Master's and Doctoral Postgraduate Program in Rehabilitation Sciences of the Nove de Julho University (Brazil) (Figure 1). The design, conduction and reporting of this study will follow the norms of the "Standards for the Reporting of Diagnostic accuracy studies" - STARD statement.

### Subjects

Male professional bus drivers of an interstate roadway firm will be invited to participate in the study. The community-based sample includes shift workers: individuals working regular night shifts (from 10:00 pm to 08:00 am), individuals working regular day shifts (from 08:00 am to 06:00 pm), and subjects working rotating shifts.

With regard to the inclusion criteria, the sample will be made up by medium and long distance drivers, agreeing to participate in the study through a signed informed consent form. Subjects with acute pulmonary or heart disease and/or major cranio-facial or upper airway abnormalities, drug users and alcoholics will be excluded from the study.

### Protocol

The initial population of professional bus drivers includes 710 subjects. All will be asked to be part of the study and all procedures will be clarified. Those who agree to participate will undergo a detailed patient history, physical examination involving determination of blood pressure, anthropometric data, circumference measurements (hips, waist and neck), tonsils and Mallampati index. Moreover, specific questionnaires addressing sleep apnea and excessive daytime sleepiness will be administered. Data acquisition will be completely anonymous. Following the medical examination, the participants will perform a spirometry, NEP test and standard overnight polysomnography.

### Physical examination

Weight and height evaluation will be performed through an electronic balance (model 200/5, Welmy Industria e Comercio Ltda, Sao Paulo, Brazil) and body mass index (BMI) will be calculated [33]. For the assessments of tonsils and Mallampati index, subjects will be in the sitting position and instructed to open the mouth as much as possible [34,35].

Waist circumference will be measured with the subject in the upright position, standing at the midpoint between the costal margin and the iliac crest at the end of normal expiration. The hip circumference will be measured with the subject in the same position at the level of the greater trochanter. Neck circumference will be measured with the subject in the sitting position at the edge of the cricoid cartilage.

### OSA and excessive daytime sleepiness questionnaires

The Berlin Questionnaire will be administered. This 10-item questionnaire has recognized efficacy in distinguishing subjects at greater risk for OSA in primary care population [36]. The Epworth Sleepiness Scale [37] will be used for the assessment of excessive daytime sleepiness. Moreover, a novel OSA questionnaire designed by researchers of the Italian National Research Council - Institute of Biomedicine and Molecular Immunology "A. Monroy" and Centro Medico di Veruno will be administered.

### Spirometry

The spirometry will be carried out during the day, with the patient seated in a comfortable position. For such, the KoKo PFT System Version 4.11 (nSpire Health, Inc; Louisville, CO, USA) will be used following national guidelines for the execution of lung function tests by the Brazilian Society of Pneumology [38] and the European Respiratory Society [39]. The subjects will perform the test in a comfortable position, with the body erect and the upper limbs unsupported. All examinations will be carried out by a competent technician trained in obtaining the necessary cooperation from the subjects and appropriately operating the equipment in order to ensure accurate, reproducible results. The equipment will be calibrated prior to each exam with a 3-L syringe [38].

### Negative expiratory pressure - NEP test

The NEP test is performed through the administration of negative pressure at the mouth during expiration. This is a practical test performed while awake and requires little cooperation from the subject. In the absence of expiratory flow limitation, the increase in the pressure gradient between the alveoli and open upper airway caused by NEP results in an increase in expiratory flow.

**Figure 2:**
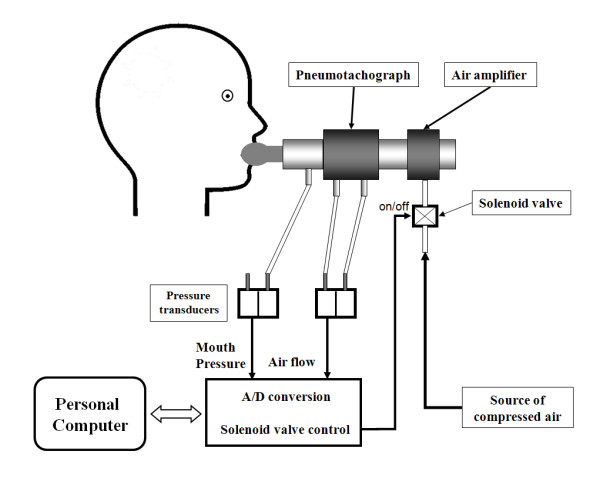
**Schematic representation of the negative expiratory pressure apparatus**.

NEP will be generated by a Super Air Amplifier (Exair model 120021 Cincinnati, Ohio, USA) coupled to a tank of compressed air via an electrically operated solenoid valve (Norgren Ltd model 95004; Vimercate, MI, Italy) automatically activated in early expiration and kept open for 2 s by software control (Figure 2). A pneumotachograph (Hans Rudolph model 3830; Kansas City, MO, USA) will be connected to the air amplifier and the mouthpiece to measure airflow ($\dot{\text{V}}$) with pressure transducers (PCLA02X5; Sensortechnics GmbH, Puchheim, Germany). Mouth pressure will be measured by pressure transducers (PCLA0050; Sensortechnics GmbH, Puchheim, Germany). NEP of 10 cm H_2_O will be set by occluding the pneumotachograph with a stopper and adjusting the flow of compressed air to air amplifier (Figure 2).

NEP maneuvers will be performed (each after at least four breaths to normalize the breathing pattern). The tests will be carried out once with the subjects seated comfortably and one other in supine position on a cot. In both positions, care will be taken to maintain the subjects comfortable with the neck in a neutral position, as it has been documented that the position of the head exerts an influence over upper airway collapsibility [40]. All maneuvers will be performed with the subjects awake and wearing a nose clip. The airflow and the mouth pressure signals will be low-pass filtered and sampled at 200 Hz. Both digital signals will be displayed in real time on the monitor and stored on the computer for subsequent analysis. Signal analysis and solenoid valve control will be performed using software written in Labview 8.2 (National Instruments) developed by the Italian National Research Council, Institute of Biomedicine and Molecular Immunology "A. Monroy".

**Figure 3:**
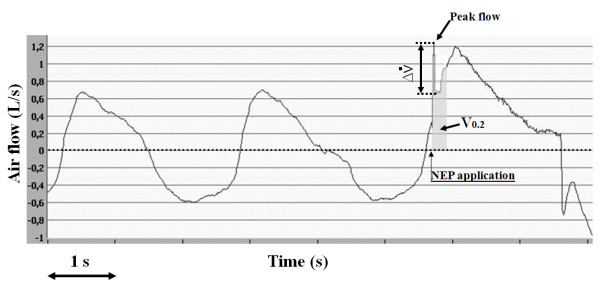
**Measurements techniques for upper airway collapsibility evaluated as expiratory volume in 0.2s (V_0.2 _- percent of the mean inspiratory volume of the three breaths preceding NEP application) and as the flow drop (Δ$\dot{\text{V}}$- expressed as the percent of the peak flow)**.

NEP application during tidal expiration produces an immediate peak flow followed by a sudden drop of a variable degree. Upper airway collapsibility is evaluated by measuring flow limitation as flow drop (Δ$\dot{\text{V}}$), expressed as the percentage of peak flow immediately after NEP administration. To avoid reflex and voluntary reactions to the NEP stimulus, the minimal flow will be identified in the first 200 ms of NEP administration [41]. Upper airway collapsibility is also evaluated by measuring V_0.2 _immediately after NEP administration (Figure 3). These values are expressed as the percentage of mean inspiratory volume of the three breaths preceding NEP administration. Measured volumes are accepted only when differences between inspiration and expiration for each of the three previous breaths are less than 10%. Values of V_0.2 _and Δ$\dot{\text{V}}$ (%) are calculated as the mean of four measurements.

### Standard overnight polysomnography

Nocturnal polysomnography will be carried out using the Embla Somnologica Studio - EMBLA A10, version 3.1.2. (Flaga hf. Medical Devices, Iceland) sleep analysis system. The following signals will be recorded: electroencephalogram (F4-M1, C4-M1, O2-M1, and backup electrodes F3, C3, O1 and M2), electrooculograms (E1-M2, E2-M2), chin and tibials electromyography, electrocardiogram, oxygen saturation, pulse rate, oronasal airflow (nasal canula and thermistor), respiratory effort (thoracic and abdominal Xtrace model straps), snoring, and body position. The patients will be instructed to remain relaxed and sleep naturally as at home. Throughout the night, the subjects will be monitored by a technician experienced in polysomnography [42]. The reading will be performed by professionals who were blinded to the result of the NEP test, following the guidelines of the American Academy of Sleep Medicine [43] and criteria of the Brazilian Sleep Society.

Apneas are defined as lack of airflow or a reduction ≥90% in the airflow signal for at least 10 s. Hypopneas are defined as discernible drop in flow by ≥ 30% of baseline for a period lasting at least 10 s followed by a SaO_2 _fall ≥4%. AHI will be calculated as number of (apneas + hypopneas)/h of total sleep time. Subjects with AHI ≥5 will be classified as OSA [42,43].

### Quality control

In order to ensure data quality, the physiotherapists and physicians in charge of data collection will receive specific training. Periodic external monitoring will be performed to verify adequate application of methodology in performing examinations and data collection.

### Calculation of sample size and statistical analysis

The calculation of sample size was based on the correlation reported by Insalaco et al. [44] between Δ$\dot{\text{V}}$(%) and AHI in patients with OSA (r = 0.37). For a bidirectional alpha value of 0.05 and 90% power, a minimum number of 82 subjects will be necessary.

Kolmogorov-Smirnov normality test will first be performed in order to determine the presence or absence of a normal distribution sample. Descriptive analysis will be performed, with the data expressed as either mean and standard deviation or median values and 95% confidence intervals, when appropriate. One-way analysis of variance (ANOVA) will be used for comparisons between work shifts once the samples have a normal distribution. V_0.2 _and Δ$\dot{\text{V}}$(%) values will be linearly correlated with the AHI, for which either Pearson's or Spearman's correlation test will be used, depending on the sample distribution. Either the non-paired Student's t-test or Mann-Whitney test will be used for comparisons between individuals with and without OSA. Logistic regression for the analysis of continuous factors with categorical responses will be performed. Receiver operating characteristic (ROC) curves will be constructed to determine the sensitivity (true positive rate) versus 100-specificity (false positive rate) at various levels of the measured Δ$\dot{\text{V}}$ (%) and V_0.2 _(%) to identify the cut-off value yielding the largest number of correctly classified patients. The statistical analysis will be performed by an experienced statistician using the JMP commercial program (version 8.0, SAS Institute Inc.) and SPSS program (version 16.0, Somers NY). A 5% level of significance and 95% confidence interval will be applied.

### Ethical considerations

The present study is in accordance with the Helsinki Declaration and the Regulatory Guidelines and Norms for Research Involving Human Subjects of the National Health Board of the Brazilian Health Ministry issued in October 1996. This study received approval from the Human Research Ethics Committee of the Nove de Julho University (Brazil) under process number 329445/2010. Informed consent will be required to all subjects.

All procedures of the study will be confidential. The professional drivers diagnosed for OSA will be referred to the Sleep Medicine service and immediately forwarded to adequate treatment in order to treat the sleep respiratory disorder.

## Discussion

Despite the abundance of scientific evidence, OSA is still underdiagnosed in the general population. This is probably due to multiple causes, such as deficiency of knowledge on the part of physicians and also the limited access of the patients to diagnosis and treatment of OSA [45]. In addition, diagnostic procedures are expensive, and predictive criteria are still unsatisfactory. Obesity parameters are important predictors, although not all OSA patients are obese and not all obese subjects have OSA. The identification of new markers of OSA would be useful.

The aim of the present protocol study is to determine whether the NEP test could be used as a screening tool for OSA and the prevalence of this condition among a population of professional interstate bus drivers. Because increased upper airway collapsibility is one of

the main determinants of OSA [21], the response to the application of NEP could be a predictor of this disorder.

With the enrollment of this study protocol, the expectation is to encounter predictive NEP values for different degrees of OSA. It's a daytime, low-cost screening method that could be included in a preventive strategy in order to contribute toward an early diagnosis of this condition and reduce its impact and complications among professional interstate bus drivers.

## Competing interests

The authors declare that they have no competing interests.

## Authors' contributions

LVFO, GI and RPH provided the idea for the study, established the hypothesis and wrote the original proposal. SR, GI and AS developed the NEP system and software used in this protocol. RPH, IRS, ICA, NSFJ, LCG, PNN and SRN took part in the data collection. ICA, NSFJ, CSO, PTC and IRS participated in the organization and reporting of the data. FSSLF, LMMS, SR, LCG and SRN worked on the data collection, statistical analysis, evaluation and presentation of the results. RPH, AB and LVFO significantly contributed to writing this protocol paper with the input of all co-authors, while GI, AS, FSSLF, GLF and LMMS were involved in critically revising the manuscript. All authors read and approved the final manuscript.

## Pre-publication history

The pre-publication history for this paper can be accessed here:

http://www.biomedcentral.com/1471-2466/11/57/prepub
